# Systolic and diastolic dysfunction affects kidney outcomes in hospitalized patients

**DOI:** 10.1186/s12882-018-1103-2

**Published:** 2018-10-23

**Authors:** Jae Shin Choi, Seon Ha Baek, Ho Jun Chin, Ki Young Na, Dong-Wan Chae, Yon Su Kim, Sejoong Kim, Seung Seok Han

**Affiliations:** 1 Department of Internal Medicine, Hana General Hospital, Cheongju-si, Chungcheongbuk-do South Korea; 2 Department of Internal Medicine, Hallym University Dongtan Hospital, Gyeonggi-do, South Korea; 3 Department of Internal Medicine, Seoul National University College of Medicine, 103 Daehakro, Jongno-gu, Seoul, 03080 South Korea; 4 Department of Internal Medicine, Seoul National University Bundang Hospital, 82, Gumi-ro 173beon-gil, Bundang-gu, Seongnam-si, Gyeonggi-do 13620 South Korea

**Keywords:** Acute kidney injury, Echocardiography, End-stage renal disease, Diastolic dysfunction, Systolic dysfunction

## Abstract

**Backgrounds:**

Knowledge on cross-talk between the heart and kidney has been established by basic and clinical research. Nevertheless, the effects of systolic and diastolic heart dysfunctions on the development of acute kidney injury (AKI) and end-stage renal disease (ESRD) remain unresolved in hospitalized patients.

**Methods:**

A total of 1327 hospitalized patients who had baseline transthoracic echocardiography performed were retrospectively analyzed. Patients were categorized by the quartiles of ejection fraction (EF) and the ratio of the early transmitral blood flow velocity to early diastolic velocity of the mitral annulus (E/e’). The odds ratios (ORs) for AKI and the hazard ratios (HRs) for ESRD were calculated after adjustment of multiple covariates.

**Results:**

During hospital admission, AKI occurred in 210 (15.8%) patients. The lowest quartile of EF was associated with a risk of AKI (OR, 1.60 [1.07–2.41]) and the highest quartile of E/e’ was associated with a risk of AKI (OR, 1.90 [1.26–2.41]). When two echocardiographic parameters were combined, patients with a low EF (first to second quartiles) and high E/e’ (fourth quartile) showed the highest OR for AKI (OR, 2.27 [1.49–3.45]) compared with the counterpart patients. When the risk of ESRD was evaluated, E/e’, but not EF, was a significant parameter of high risk (fourth vs. first quartiles: HR, 4.13 [1.17–14.64]).

**Conclusions:**

Baseline systolic and diastolic dysfunction is related to subsequent risks of AKI and ESRD in hospitalized patients. Monitoring of these parameters may be a useful strategy to predict the risk of these adverse events in the kidney.

## Background

Despite advances in medical practice, the incidence of acute kidney injury (AKI) and its related mortality are still high as approximately 25% in adults [1]. The clinical implication is equal to those of chronic kidney disease (CKD). The prevalence of CKD ranges from 5 to 13% [2, 3], and its related risk of mortality is five times higher than that in non-CKD patients [4]. The problems become further serious in the cases of end-stage renal disease (ESRD), the last stage of AKI and CKD. Hospitalization rates in patients with ESRD have increased within the last 10 years, with increases by 40% form infections, 200% form vascular access problems, and 30% form cardiovascular diseases [5]. Collectively, both AKI and CKD are related to several morbidities and high mortality. Accordingly, it is needed to characterize the risk factors related with these kidney outcomes in clinical practice.

The heart and kidney are functionally related each other. If one of these two organs has dysfunction, other organs will also experience dysfunction. A close relationship between these two organs (heart-kidney) is referred to as the phenomenon of cardiorenal syndrome (CRS). The clinical significance of CRS has been the focus in several clinical cases, including ST-segment elevation myocardial infarction [6], coronary artery bypass surgery [7], and sepsis [8]. Nevertheless, this issue has been unresolved in overall hospitalized patients in whom the causes of admission are heterogeneous. Therefore, the present study aimed to investigate systolic and diastolic function of the heart using echocardiography at baseline, and examined its relationship with the risk of kidney dysfunction including AKI and ESRD.

## Methods

### Data source and study samples

The main data source used in this study was obtained from a cohort of patients from Seoul National University Bundang Hospital. A total of 21,574 patients were admitted in this hospital from January 2013 to December 2013. We included patients who underwent an echocardiography examination with tissue Doppler image before hospitalization. Finally, 1327 patients were analyzed in this study.

### Demographic, medical, and laboratory data

Demographic and medical data including age, sex and comorbid conditions were obtained from medical records or interviews with patients. The body mass index was calculated as the ratio of weight in kilograms divided by square of height in meters. Data on hemoglobin, serum albumin, fasting glucose, cholesterol level were collected. The baseline value of the estimated glomerular filtration rate (eGFR) was calculated using the Chronic Kidney Disease Epidemiology Collaboration equation [9]. Additionally, information regarding patients’ medications, including aspirin, angiotensin-converting enzyme inhibitors, angiotensin II receptor blockers, alpha/beta-blockers, calcium channel blockers, and diuretics during the study period was obtained from medical records.

AKI was defined as an increase in serum creatinine by ≥0.3 mg/dl or ≥ 1.5 times above baseline during hospital admission, in adherence to the Kidney Disease Improving Global Outcomes guideline [10]. ESRD was defined as commencement of dialysis. All-cause mortality data were obtained from the national database of Statistics Korea. The patients were followed until August 2015, except for the death-censored cases.

### Echocardiographic data

Echocardiograms were performed within 1 year before the date of admission using standard techniques to evaluate cardiac function in lateral decubitus position. Left ventricular ejection fraction (LVEF) was calculated using biplane approach and modified Simpson method from apical imaging planes [11]. The left ventricular mass index was calculated using the method of Devereux et al. [12]. In the apical 4-chamber view, early (E) and late (A) transmitral inflow velocities and early (e’) and late (a’) diastolic mitral annular peak velocities were measured by pulsed-wave spectral Doppler tissue images from the same view on the septal side of the mitral annulus.

### Statistical analysis

Statistical analyses ware performed using SPSS version 20.0 (IBM, Armonk, NY, USA). Data are presented as percentages for categorical parameters. Mean (± standard deviations) or median (interquartile ranges) was used for continuous parameters. Odds ratios (ORs) and confidence intervals for AKI according to the echocardiographic parameters were calculated using logistic regression analysis. Survival curves were expressed using Kaplan-Meier method. The Cox proportion hazard model was used to calculate the hazard ratio (HR) of ESRD risk according to the echocardiographic parameters. Comparisons between non-normally distributed continuous variables were performed using the Mann-Whitney U test. Multiple comparisons among the study groups were performed by Kruskal-Wallis test followed by a post hoc test that was adjusted with a less significant difference correction. The discrimination of predicting outcomes by echocardiographic parameters was assessed by calculating the area under the receiver operating characteristic curve. Model performance was additionally assessed using the continuous net reclassification improvement (cNRI) and integrated discrimination improvement (IDI). Both of cNRI and IDI were analyzed by using R software (version 3.4.4; The Comprehensive R Archive Network: http://cran.r-project.org). A difference was considered significant if the value was less than 0.05.

## Results

### Baseline characteristics

A total of 1327 patients were included in this study and 52.4% were men. The mean age of the patients was 66.2 ± 13.4 years and mean eGFR was 82.5 ± 24.1 ml/min/1.73 m^2^ at baseline. A total of 210 (15.8%) patients developed AKI during admission. The patients’ demographic, medical, and laboratory data, and echocardiographic parameters are shown in Table 1. Multiple variables in baseline characteristics, such as hypertension, diabetes mellitus, and heart failure, were associated with AKI. Among the echocardiographic parameters, LVEF and E/e’ were associated with AKI.

**Table 1:** Baseline characteristics of the study patients

Variable	Total (*n* = 1327)	Non-AKI (*n* = 1117)	AKI (*n* = 210)	*P*
Age (year)	66.2 ± 13.4	66.1 ± 0.4	66.9 ± 1.1	0.104
Men (%)	52.4	52.8	50.0	0.453
Body mass index (kg/m^2^)	24.2 ± 3.7	24.3 ± 0.1	23.2 ± 0.3	< 0.001
Systolic BP (mmHg)	129.5 ± 20.0	130.3 ± 0.6	125.5 ± 1.6	0.001
Diastolic BP (mmHg)	73.6 ± 12.7	74.3 ± 0.4	70.3 ± 1.0	< 0.001
Comorbidities (%)				
Hypertension	42.0	40.5	50.0	0.010
Diabetes mellitus	32.4	28.6	52.4	< 0.001
Ischemic heart disease	21.1	23.3	9.0	< 0.001
Cerebrovascular disease	6.1	6.2	5.7	0.797
Heart failure	2.1	1.5	5.2	0.001
Cancer	31.3	29.8	39.5	0.005
Medications (%)				
Alpha blocker	0.9	0.6	2.4	0.014
ACE inhibitor	5.3	4.8	7.6	0.098
ARB	17.9	17.0	22.4	0.062
Beta-blocker	18.6	19.2	15.7	0.239
Calcium channel blocker	16.5	14.8	25.7	< 0.001
Diuretics	14.0	9.2	4.8	< 0.001
Laboratory findings				
Hemoglobin (g/dL)	12.2 ± 2.1	12.4 ± 0.06	10.9 ± 0.1	< 0.001
Albumin (g/dL)	3.9 ± 0.6	3.9 ± 0.02	3.5 ± 0.04	< 0.001
Glucose (mg/dL)	134.3 ± 56.8	130.4 ± 1.6	149.6 ± 4.8	< 0.001
Cholesterol (mg/dL)	157.3 ± 41.6	160.3 ± 1.2	141.6 ± 3.3	< 0.001
eGFR (ml/min/1.73 m^2^)	82.5 ± 24.1	83.0 ± 20.6	79.9 ± 37.7	0.376
Echocardiographic findings				
LVMI (g/m^2^)	95.8 ± 26.5	95.3 ± 0.8	98.5 ± 2.1	0.220
EF (%)	61.6 ± 8.8	61.9 ± 0.3	60.3 ± 0.7	0.027
E/A	0.9 ± 0.4	0.9 ± 0.01	0.9 ± 0.03	0.228
E/e’	10.3 ± 4.9	10.1 ± 0.1	11.6 ± 0.4	0.001
RWMA (%)	8.8	8.6	10.0	0.510

*AKI* acute kidney injury, *BP* blood pressure, *ACE* angiotensin-converting enzyme, *ARB* angiotensin II receptor blocker, *eGFR* estimated glomerular filtration rate, *LVMI* left ventricular mass index, *EF* ejection fraction, *E* early diastolic transmitral inflow velocity, *A* late diastolic transmitral flow velocity, *e’* early diastolic mitral annular velocity, *RWMA* regional wall motion abnormality

### AKI according to echocardiographic parameters

**Fig. 1:**
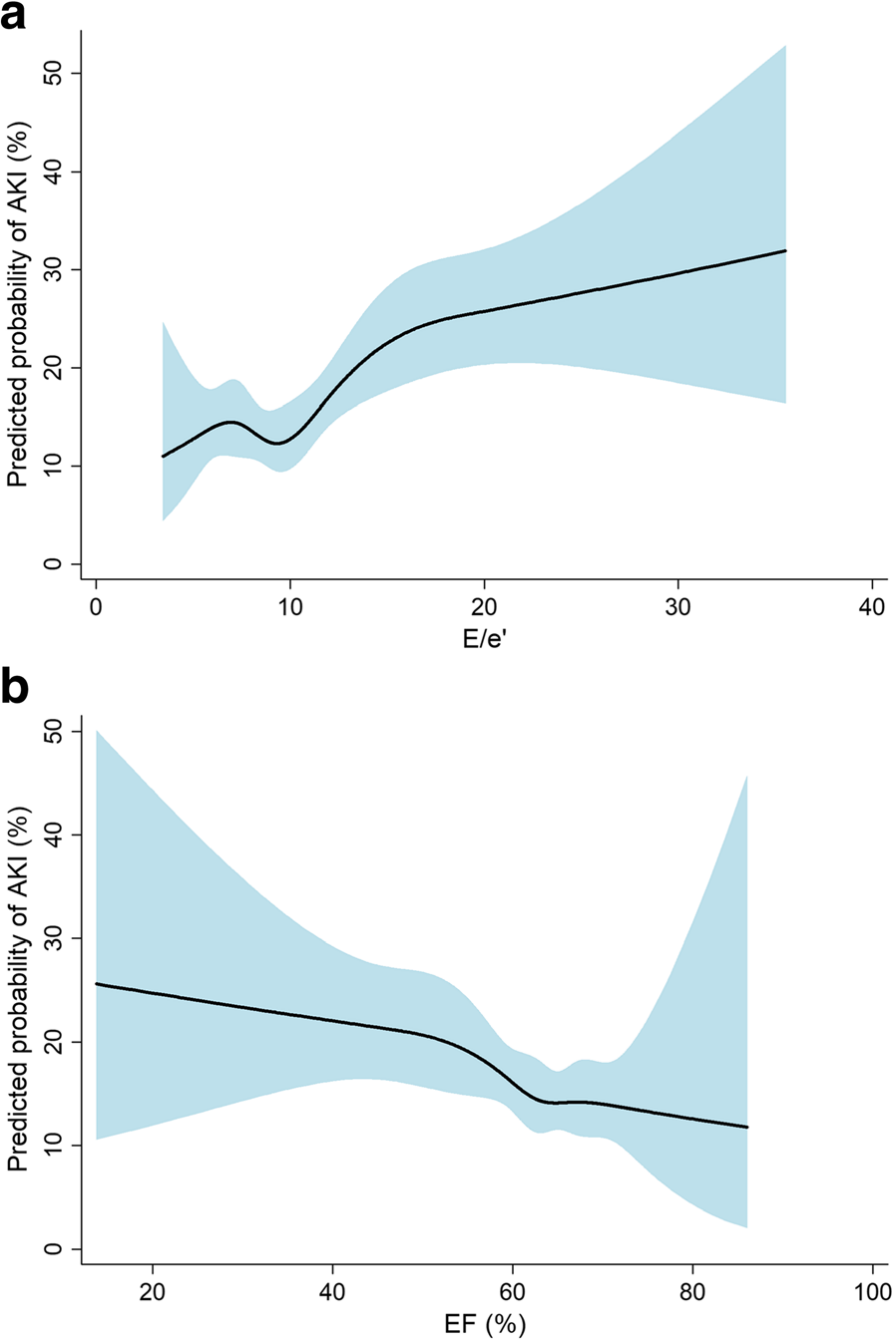
Nonlinear relations between predicted probability of AKI and echocardiographic parameters, (**a**) E/e’ and (**b**) LVEF. Fitted line and 95% confidence intervals are indicated as solid and shaded area, respectively

Table 2 shows the ORs of AKI according to the echocardiographic parameters. LVEF and E/e’ were categorized by quartiles of each parameter. Additionally, combined parameter was defined using the median of LVEF and quartile of E/e’. When baseline clinical and laboratory parameters and other echocardiographic parameters were adjusted, the first quartile of LVEF and the fourth quartile group of combined parameters were selected as independent predictors of AKI (all *P*s <  0.05). E/e’ showed a tendency for an association with AKI, with a tendency of an increasing OR value. Based on the non-linear relationship curves, a greater E/e’ and smaller LVEF were associated with high risk of AKI (Fig. 1).

**Table 2:** Odds ratios of acute kidney injury according to the echocardiographic parameters

			Univariate		Multivariate^a^	
Parameters		Range	OR (95% CI)	*P*	OR (95% CI)	*P*
Ejection fraction	4th quartile (*n* = 355)	> 67.1	1 (Reference)		1 (Reference)	
	3rd quartile (*n* = 322)	63.1–67.1	1.07 (0.693–1.644)	0.768	1.08 (0.663–1.748)	0.765
	2nd quartile (*n* = 322)	58.5–63.0	1.08 (0.705–1.658)	0.720	1.27 (0.788–2.039)	0.328
	1st quartile (*n* = 318)	< 58.5	1.60 (1.069–2.409)	0.023	1.74 (1.035–2.917)	0.037
E/e’	1st quartile (*n* = 328)	< 7.2	1 (Reference)		1 (Reference)	
	2nd quartile (*n* = 338)	7.2–9.2	1.12 (0.723–1.747)	0.604	1.15 (0.699–1.888)	0.583
	3rd quartile (*n* = 338)	9.3–11.9	1.04 (0.668–1.632)	0.850	1.06 (0.635–1.777)	0.817
	4th quartile (*n* = 323)	> 11.9	1.90 (1.257–2.877)	0.002	1.63 (0.954–2.787)	0.074
Combined parameter^b^	1st group (*n* = 537)	–	1 (Reference)		1 (Reference)	
	2nd group (*n* = 466)	–	1.20 (0.836–1.719)	0.325	1.33 (0.895–1.977)	0.158
	3rd group (*n* = 138)	–	1.68 (1.026–2.743)	0.039	1.49 (0.852–2.612)	0.162
	4th group (*n* = 186)	–	2.27 (1.490–3.446)	< 0.001	1.95 (1.192–3.201)	0.008

^a^Adjusted for age, sex, body mass index, comorbidities, medications, laboratory findings, and other echocardiography findings

^b^Combined parameter: 1st group, 1st to 3rd quartiles of E/e’ plus 3rd to 4th quartiles of ejection fraction; 2nd group, 1st to 3rd quartiles of E/e’ plus 1st to 2nd quartiles of ejection fraction; 3rd group, 4th quartile of E/e’ plus 3rd to 4th quartiles of ejection fraction; 4th group, 4th quartile of E/e’ plus 1st to 2nd quartiles of ejection fraction

*OR* odds ratio, *CI* confidence interval, *E* early diastolic transmitral inflow velocity, *A* late diastolic transmitral flow velocity, *e’* early diastolic mitral annular velocity

### ESRD according to echocardiographic parameters

**Fig. 2:**
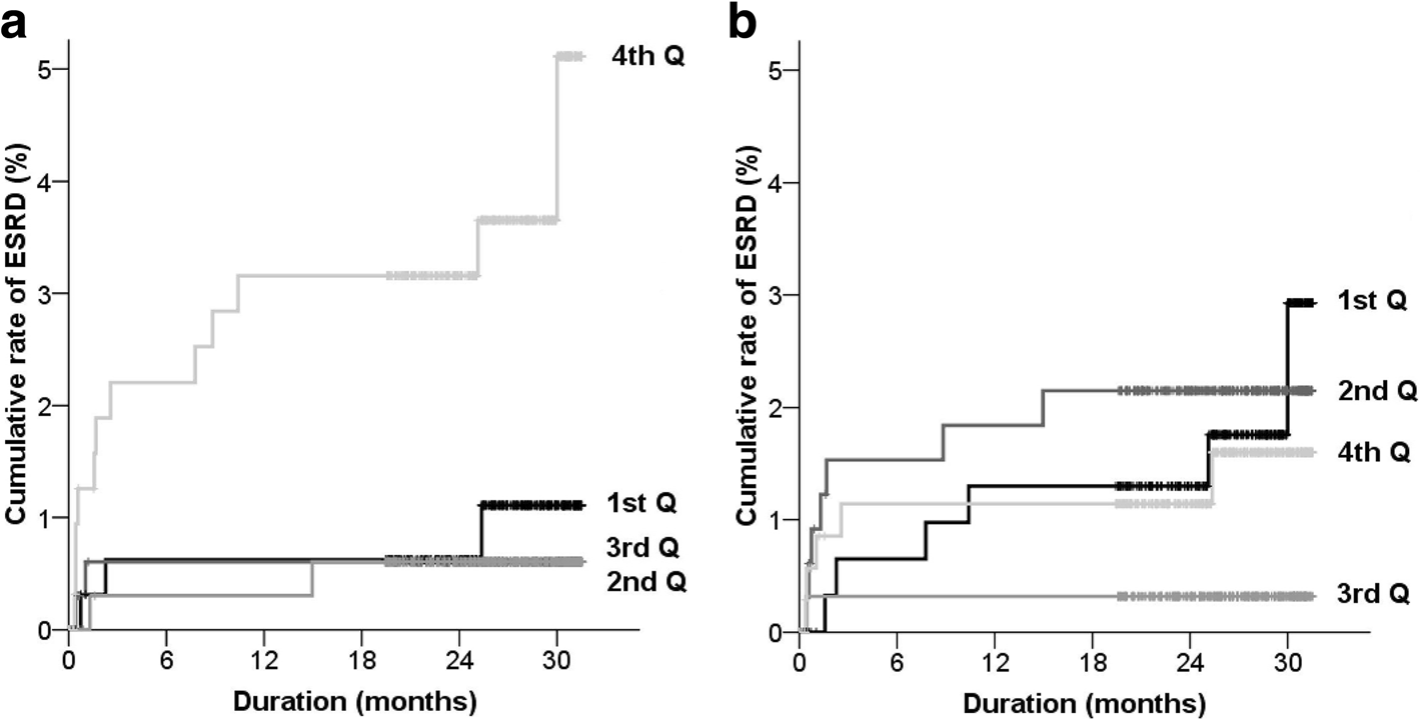
Curves obtained using the Kaplan-Meier method of rates of ESRD according to groups. **a** E/e’ and (**b**) LVEF

Figure 2 shows Kaplan-Meier curves of ESRD based on the echocardiographic parameters. The highest E/e’ group seemed to be at the high risk of ESRD compared with other E/e’ groups, whereas EF was not associated with the risk trend of ESRD. Table 3 shows HRs of ESRD according to the echocardiographic parameters. In univariate analysis, the fourth quartile of E/e’ had a risk of ESRD. When combined parameter was used, the group with low LVEF and high E/e’ showed a tendency of increased risk of ESRD in univariate analysis. However, there were reduced significances between ERSD and echocardiographic parameters in multivariate analysis (Table 3).

**Table 3:** Hazard ratios of end-stage renal disease according to the echocardiographic parameters

		Univariate		Multivariate^a^	
Parameters		HR (95% CI)	*P*	HR (95% CI)	*P*
Ejection fraction	4th quartile (*n* = 355)	1 (Reference)		1 (Reference)	
	3rd quartile (*n* = 322)	0.22 (0.026–1.881)	0.167	0.26 (0.067–1.027)	0.055
	2nd quartile (*n* = 322)	1.51 (0.478–4.749)	0.484	0.79 (0.226–2.746)	0.708
	1st quartile (*n* = 318)	1.33 (0.405–4.348)	0.641	0.12 (0.013–1.093)	0.060
E/e’	1st quartile (*n* = 328)	1 (Reference)		1 (Reference)	
	2nd quartile (*n* = 338)	0.64 (0.107–3.828)	0.624	2.31 (0.171–31.098)	0.528
	3rd quartile (*n* = 338)	0.64 (0.106–3.806)	0.620	0.94 (0.076–11.629)	0.961
	4th quartile (*n* = 323)	4.13 (1.165–14.635)	0.028	5.20 (0.548–49.273)	0.151
Combined parameter^b^	1st group (*n* = 537)	1 (Reference)		1 (Reference)	
	2nd group (*n* = 466)	1.55 (0.347–6.930)	0.566	1.44 (0.234–8.819)	0.695
	3rd group (*n* = 138)	4.09 (0.825–20.258)	0.085	4.02 (0.517–31.282)	0.183
	4th group (*n* = 186)	8.81 (2.386–32.554)	0.001	3.57 (0.596–21.340)	0.164

^a^Adjusted for age, sex, body mass index, comorbidities, medications, laboratory findings, and other echocardiography findings

^b^Combined parameter: 1st group, 1st to 3rd quartiles of E/e’ plus 3rd to 4th quartiles of ejection fraction; 2nd group, 1st to 3rd quartiles of E/e’ plus 1st to 2nd quartiles of ejection fraction; 3rd group, 4th quartile of E/e’ plus 3rd to 4th quartiles of ejection fraction; 4th group, 4th quartile of E/e’ plus 1st to 2nd quartiles of ejection fraction

*HR* hazard ratio, *CI* confidence interval, *E* early diastolic transmitral inflow velocity, *A* late diastolic transmitral flow velocity, *e’* early diastolic mitral annular velocity

**Fig. 3:**
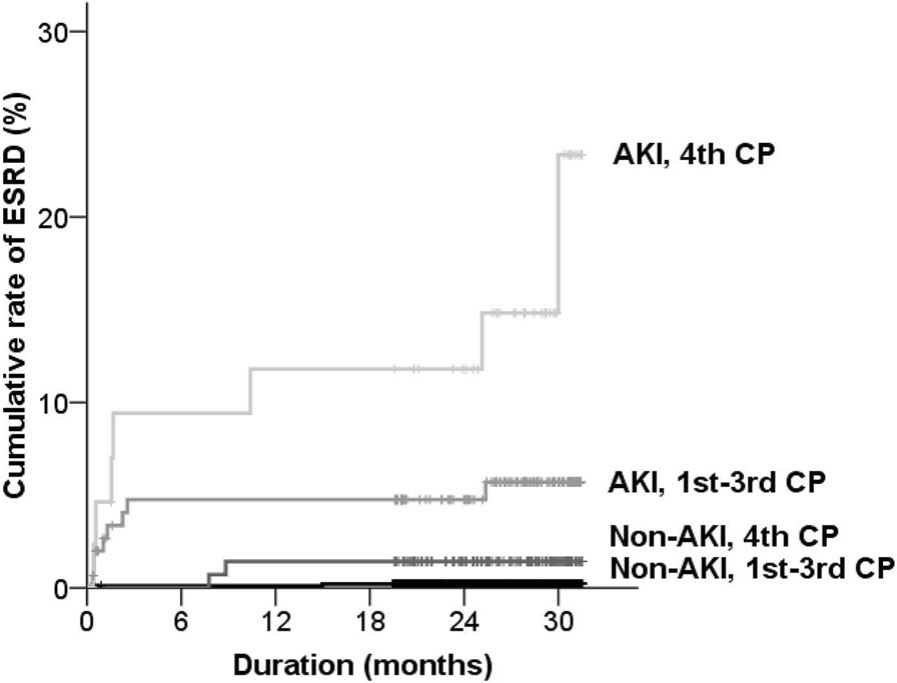
Risk of ESRD according to the presence of AKI and heart dysfunction. Combined parameter (CP) is defined using the median of LVEF and quartiles of E/e’, as follows: 1st group, 1st to 3rd quartiles of E/e’ plus 3rd to 4th quartiles of ejection fraction; 2nd group, 1st to 3rd quartiles of E/e’ plus 1st to 2nd quartiles of ejection fraction; 3rd group, 4th quartile of E/e’ plus 3rd to 4th quartiles of ejection fraction; 4th group, 4th quartile of E/e’ plus 1st to 2nd quartiles of ejection fraction

Figure 3 shows the risk of ESRD according to the presence of AKI and heart dysfunction. The risk of ESRD was assessed by dividing the groups with and without AKI and by the first to third combined parameter group and fourth combined parameter group. The non-AKI and fourth combined parameter group (HR, 5.95 [0.373–95.107]; *P* = 0.207), the AKI and first to third combined parameter group (HR, 29.47 [2.499–347.649]; *P* = 0.007), and the AKI and fourth combined parameter group (HR, 31.31 [2.544–385.238]; *P* = 0.007) had higher HRs compared with the non-AKI and first to third combined parameter group. These results indicated that heart dysfunction affected the risk of ESRD separately by the presence of AKI.

### Discriminant analysis and risk reclassification analysis

The areas under the receiver operating characteristic curves (AUCs) predict AKI was 0.548 (0.505–0.591) for LVEF and 0.570 (0.526–0.614) for E/e’. The AUCs of 3-year ESRD were 0.566 (0.426–0.706) and 0.690 (0.544–0.836) for LVEF and E/e’, respectively. We evaluated whether the addition of echocardiographic parameters to risk models increased overall predictability for AKI and 3-year ESRD. The reference risk model was composed of covariates that had been known as a risk factor of cardiovascular diseases in addition to age and sex. Table 4 shows the AUCs for the risk models that predict AKI and ESRD. Compared with the reference model, the AUC of the risk model including LVEF and E/e’ improved predictive capacity of AKI from 0.695 (0.657–0.733) to 0.709 (0.672–0.745) (*P* = 0.027). When model performance was assessed using the cNRI and IDI indices, the risk models including E/e’ (model 2) or E/e’ and EF together (model 3) had an improved predictability of outcomes compared with the reference model (Table 5). Collectively, the overall predictability of AKI and ESRD was improved when echocardiographic parameters were additionally considered.

**Table 4:** Comparison between the areas under the receiver operating characteristic curves of echocardiographic models

	Reference	Model 1		Model 2		Model 3	
	AUC (95% CI)	AUC (95% CI)	P*	AUC (95% CI)	P*	AUC (95% CI)	P*
AKI	0.695 (0.657–0.733)	0.705 (0.668–0.742)	0.065	0.703 (0.665–0.740)	0.161	0.709 (0.672–0.745)	0.027
3-year ESRD	0.788 (0.669–0.907)	0.795 (0.677–0.913)	0.536	0.819 (0.686–0.951)	0.204	0.819 (0.688–0.951)	0.182

Reference model: age, sex, body mass index, diabetes, ischemic heart disease, and cerebrovascular disease

Model 1: reference model plus ejection fraction

Model 2: reference model plus E/e’

Model 3: reference model plus combined parameter

*Difference is calculated in comparison to the reference model

*AUC* area under the curve, *CI* confidence interval, *AKI* acute kidney injury, *ESRD* end-stage renal disease

**Table 5:** Continuous NRI and IDI indices when the echocardiographic parameters are added in the AKI- or ESRD-predicting model

Outcome	Parameter	cNRI (95% CI)	P*	IDI (95% CI)	P*
AKI	Model 1	0.109 (− 0.038–0.256)	0.145	0.008 (0.000–0.002)	0.053
	Model 2	0.211 (0.065–0.356)	0.005	0.001 (0.000–0.002)	0.013
	Model 3	0.148 (0.000–0.295)	0.049	0.002 (0.001–0.004)	0.001
3-year ESRD	Model 1	0.283 (−0.154–0.720)	0.204	0.000 (−0.000–0.001)	0.423
	Model 2	0.477 (0.077–0.876)	0.019	0.002 (0.000–0.003)	0.008
	Model 3	0.685 (0.314–1.055)	< 0.001	0.003 (0.001–0.005)	< 0.001

Reference model: age, sex, body mass index, diabetes, ischemic heart disease, and cerebrovascular disease

Model 1: reference model plus ejection fraction

Model 2: reference model plus E/e’

Model 3: reference model plus combined parameter

*Difference is calculated in comparison to the reference model. *cNRI* continuous net reclassification improvement, *IDI* integrated discrimination improvement, *AKI* acute kidney injury, *ESRD* end-stage renal disease

## Discussion

AKI and ESRD are related with morbidity and high mortality. Therefore, these pathologic conditions should be appropriately predicted and treated. The present study showed that systolic and diastolic heart dysfunction, which was reflected by LVEF and E/e’, respectively, were associated with development of AKI in hospitalized patients. Worse echocardiographic parameters showed a tendency of increased risk of ESRD. Based on the fact that heart and kidney are related each other, heart dysfunction might affect kidney function. We investigated this concept in heterogeneous hospitalized patients, not a specialized disease subset. Therefore, echocardiographic monitoring of heart dysfunction may be added to general prediction models of AKI and ESRD, and possibly to real clinical practice to predict these outcomes.

Previous studies have addressed the relationship between heart dysfunction and the risk of AKI in various clinical conditions. In patients who underwent coronary artery bypass grafting and had preserved systolic function, preoperative E/e’ > 15 was a strong independent predictor of AKI [13]. Among patients who underwent primary coronary intervention because of ST-segment elevation myocardial infarction, a high E/e’ ratio was associated with an increased risk of AKI [6]. The present study also supports these previous results, particularly in general hospitalized patients, although the cut-off value of echocardiographic parameters could be altered depending on the patients’ status.

Some studies have shown that heart dysfunction is associated with the subsequent decline in renal function and ESRD. One study determined the association of echocardiographic parameters with the rate of decline in renal function decline and progression to dialysis in CKD stage 3 to 5 patients [14]. This previous study also showed that a decreased LVEF was associated with a faster reduction of renal function. Another observational study from a regional hospital in Taiwan with 518 dialysis-independent patients with CKD stages 3 to 5 showed that left ventricular dysfunction was associated with a rapid decline in renal function [15]. A previous study that included 1045 transplant patients showed that an elevated E/e’ was associated with graft dysfunction, postoperative hemodialysis, and overall mortality [16]. Similar to the conclusions of the previous studies, a greater E/e’ seemed to be a predictor of ESRD in our study.

The above-mentioned results can be explained by the theoretical background of CRS. However, there are several types of CRS that explain the relationship between heart and kidney. The type I CRS, which is represented by a decrease in LVEF, is characterized by abrupt worsening of cardiac function of cardiac function, finally leading AKI [17]. Renal dysfunction in CRS type I is attributable to a combination of low cardiac output, which consequently causes a reduction in blood flow and renal perfusion pressure and/or venous congestion [17, 18]. Increased venous congestion causes an increase in renal interstitial pressure, which might result in a hypoxic state of kidney [17, 19]. CRS type II comprises chronic abnormalities in cardiac function (e.g. chronic congestive heart failure) causing progressive and permanent chronic kidney disease. CRS type III consists in an abrupt worsening of renal function (e.g. acute kidney ischemia or glomerulonephritis) causing acute cardiac disorder (e.g. heart failure, arrhythmia, ischemia) [17]. All types of CRS may have played a role in the present study, but there was the limitation of not being able to determine the mechanism involved because of the observational study design.

The present study has many strengths. The sample size obtained was relatively large. Additionally, baseline data regarding covariates were all available in study patients. The occurrences of AKI and ESRD were well traced in our study. However, ESRD events have not been well reviewed in other observational studies in general. Nevertheless, this study has some limitations. This was a retrospective, observational study, and may have been subject to bias such as a causal relationship. However, the original purpose of the study was to address the predictability of heart dysfunction, not a causal relationship between two parameters. Timeframe obtaining the echocardiographic data was within 1 year, wherein certain patients’ heart status might had been altered at the time of admission. We did not measure kidney biomarkers, such as neutrophil gelatinase-associated lipocalin, kidney injury molecule-1, and interleukin-18, which might interact the relationship between heart and kidney dysfunctions [20].

## Conclusions

Baseline systolic and diastolic dysfunction is related with subsequent AKI and ESRD risks in hospitalized patients. Monitoring of these parameters may be a useful strategy to predict or reduce the risk of these kidney adverse events.

## Abbreviations

A: Late diastolic transmitral flow velocity
ACE: Angiotensin-converting enzyme
AKI: Acute kidney injury
ARB: Angiotensin II receptor blocker
CKD: Chronic kidney disease
CRS: Cardiorenal syndrome
E: Early diastolic transmitral inflow velocity
e’: Early diastolic mitral annular velocity
EF: Ejection fraction
eGFR: Estimated glomerular filtration rate
ESRD: End-stage renal disease
HRs: Hazard ratios
LVEF: Left ventricular ejection fraction
LVMI: Left ventricular mass index
ORs: Odds ratios
RWMA: Regional wall motion abnormality
